# A WSN-Based Tool for Urban and Industrial Fire-Fighting

**DOI:** 10.3390/s121115009

**Published:** 2012-11-06

**Authors:** Alberto De San Bernabe Clemente, José Ramiro Martínez-de Dios, Aníbal Ollero Baturone

**Affiliations:** Robotics Vision and Control Group, University of Sevilla, Escuela Superior de Ingenieros, c/Camino de los Descubrimientos, s/n 41092 Seville, Spain; E-Mails: adesanbernabe@us.es (A.S.B.C.); aollero@cartuja.us.es (A.O.B.)

**Keywords:** fire monitoring, wireless sensor networks, pervasive computing

## Abstract

This paper describes a WSN tool to increase safety in urban and industrial fire-fighting activities. Unlike most approaches, we assume that there is no preexisting WSN in the building, which involves interesting advantages but imposes some constraints. The system integrates the following functionalities: fire monitoring, firefighter monitoring and dynamic escape path guiding. It also includes a robust localization method that employs RSSI-range models dynamically trained to cope with the peculiarities of the environment. The training and application stages of the method are applied simultaneously, resulting in significant adaptability. Besides simulations and laboratory tests, a prototype of the proposed system has been validated in close-to-operational conditions.

## 1. Introduction

Thousands of urban and industrial fires occur each year leading to the destruction of buildings and infrastructure. Fire fighting mobilizes important resources and is a very dangerous activity, which originates many casualties every year. In most cases, the lack of information on the evolution of fire and the conditions the first responders are exposed to plays an important role in these accidents. Traditionally fire status is visually estimated by skilled firefighters. In many cases, human perception errors have led to wrong decisions. Various systems have been developed using sensors (such as GPS, thermometers, gas concentrations sensors and visual/infrared cameras) carried by firefighters. The measurements and images gathered are transmitted to a monitoring and command station, where fighting decisions are taken. Most of these solutions use single-hop communications, which are affected by limited transmission ranges. To cope with it, the communication systems used by fire brigades use low frequency bands to allow longer ranges. Even in this case, the transmission range can be insufficient in complex environments, such as large buildings and basements. In addition, if firefighters are indoors, GPS will be inoperative, and the lack of information on firefighter location can lead to accidents.

In recent years, different network communication technologies have appeared. WSN are comprised of a large number of nodes with sensing, computing and wireless communication capabilities that organize themselves in networks. Low size, cost and energy consumption are among the main features of WSN technology. Despite the simplicity of single WSN nodes, cooperation provides WSN with high flexibility, robustness and tolerance to failures. These features together with battery-based power, minimal invasion and flexible deployment have motivated the extension of WSN technologies to a growing number of applications [1]. The use of WSN in fire applications has attracted interesting research efforts. However, despite the variety of approaches and methods, very few of the reported systems have been experimentally tested.

This paper describes a WSN tool for fire-fighting activities. It integrates the following functionalities: fire monitoring, firefighter monitoring and dynamic escape path computation. It also includes a robust localization method that employs RSSI-range models dynamically trained to cope with the peculiarities of the environment. The safe escape method is based on guiding: the path information is distributed in the WSN. The Base Station uses optimization tools to periodically compute minimal cost paths using updated fire measurements. These functionalities have been experimented in physical settings. Furthermore, a prototype of the proposed system has been validated in close-to-operational conditions. This paper describes the functionalities, protocols, implementation and experimental results.

The organization of the paper is as follows. Related work is briefly described in Section 2. The general description of the proposed system is in Section 3. Section 4 presents the techniques for fire and firefighter monitoring including the training-based localization method. Section 5 describes the dynamic escape path computation. Partial and validation experimental results are in Section 6. Conclusions and future work are described in the last section.

## 2. Related Work

WSN have been proposed for the automatic detection of forest fires. Traditional technologies rely on visual cameras at static locations [2] or combined with Unmanned Aerial Vehicles [3], among others. WSN provide ubiquitous detection capabilities for forest fire. In [4], a system consisting of sensors (thermo and radiation with GPS) carried by animals living in the environment is used for forest fire detection. In [5], the design and development of a WSN involving sensors and IP cameras is described. In [6], fire detection is modeled as a k-coverage problem. In [7], a WSN with nodes equipped with temperature and humidity sensors were used to detect fire and extract measurements of the fire spread. WSNs have also been proposed for forest fire monitoring. FireWxNet consists of a WSN designed to report weather conditions as well as images in fire environments [8]. The paper shows real deployment of the WSN, which is evaluated in terms of battery performance and information gathered. Despite these efforts, WSN technology still has not solved issues such as the costs of maintaining thousands of nodes deployed in forest areas and their potential pollution.

WSN have also been proposed for detection and monitoring in urban and industrial fires. In [9], a WSN-based fire detection system was proposed to ensure safety of the people working in mines. Lim *et al.* proposed an innovative framework for residential fire detection [10]. They introduced metric of interval-message-ration (IMR) and evaluated their framework using the IMR metric. In [11], a WSN-based fire detection system for large buildings is presented. It is based on dense deployments of sensor nodes that periodically check temperature/smoke concentration and report these values to a surveillance center through WSN channels. Significant efforts have been carried out to improve WSN detection and monitoring. In [12], the authors proposed the use of machine learning techniques to improve fire detection and false alarm rejection. A WSN-based system for fire monitoring based on measurements from humidity, temperature and light sensors was proposed in [13].

WSN have also been proposed for other fire fighting activities. Significant research efforts have been devoted to the computation of escape paths in emergency evacuation. Work [14] describes a distributed method in which each node is assigned with an *altitude* value that can be seen as a degree of danger. Safe paths to exits are along sensors with high altitudes to sensors with low altitudes. The work in [15] proposes a distributed method to find safe paths to an exit through a network that can contain multiple sources of danger. Exits generate positive potentials to attract the navigating user, and obstacles generate repulsive potentials. The work in [16] uses models of the progress of the hazard and of the evacuees to ensure the evacuees stay safely ahead of the hazard. Its applicability is questionable since fire/smoke spread is difficult to be predicted and requires very complex models.

Distributed guiding methods assume that nearness between nodes is related with radio hops. However, due to radio's penetration capacity, single hop neighbor nodes are not necessarily physically close. In fact, if they are in different rooms the path length can be very high. Thus, each deployed node requires having a local map of the building to prevent guiding firefighters to a dead end. Although tedious in large deployments, this assumption is feasible in permanent settings. However, it cannot be applied if the nodes are not part of the building infrastructure, as is in our problem.

Despite this high variety of methods and approaches, few of the reported works have been experimented in physical settings and very few have been validated in close-to-operational conditions.

This paper describes a WSN-based tool to increase the level of safety in fire-fighting activities. Our work is motivated by the real application and has a pragmatic approach: we are more interested in efficacy, robustness and adaptability rather than in optimality. We assume that there is no preexisting WSN in the building: firefighters deploy the WSN nodes when they enter the building. The idea of firefighters deploying the WSN was also used in [17], which developed a solution that automatically dispenses sensor nodes to achieve reliable communication and high packet reception ratio. The rest of functionalities of interest for firefighting are not analyzed in that paper.

## 3. General Overview

Most existing works rely on a preexisting WSN deployed in the building. This assumption is not realistic in our approach. Many buildings do not have WSN deployment. In others, fire could have damaged the WSN. In our problem, firefighters deploy the nodes when they enter the building. Besides practical applicability, another advantage of this approach is that the WSN deployment can be adapted to each case, *i.e.*, increasing the node density in order to enhance the monitoring of areas of interest, e.g., with victims of with flammable materials. Rapid deployment also involves some constraints. The previous information assumed by the system should be minimized and the methods should have high adaptability to changing conditions. The delay until the WSN is operative after deployment is also an issue in these systems.

In the proposed system the WSN is comprised of static nodes and mobile nodes carried by the firefighters. When the fire brigade arrives at the fire, using a map of the building they decide the deployment of static nodes. Then, the nodes are deployed by the firefighters. It is not the objective of this paper to analyze the node deployment. It could be done, for instance, using the method described in [17]. Our paper focuses on fire monitoring, firefighter localization and escape path computation. Although deployment itself is left out of the paper, we deal with the aforementioned constraints of rapid deployment such as lack of previous information and need for high adaptability and low delays.

Our system requires only two reasonable assumptions. The Base Station needs to know the building map, *i.e.*, a representation of the configuration of rooms and hallways. Each static node needs to know the location in the map where it has been deployed. None of them is a significant constraint for applicability. Building map databases are often available to emergency brigades. In addition, as soon as the deployment for a certain fire has been decided, nodes can be informed about their locations using simple configuration packets.

Static nodes provide a communication backbone with the Base Station through multi-hop routes computed dynamically to take into account the changing conditions. All nodes are equipped with suitable fire and smoke sensors. Measurements from static nodes are used for fire monitoring. Measurements from mobile nodes are used to monitor the conditions each firefighter is currently facing. The readings from all nodes are transmitted to the Base Station, typically in the fire truck, where measurements are visualized and registered.

The proposed system includes the following main functionalities:

- *Fire monitoring*. Sensor measurements are periodically gathered from static nodes and transmitted to the Base Station using multi-hop routes.
- *Firefighter monitoring*. The objective is to gather and make available at the Base Station updated measurements of each firefighter including its location and the conditions the firefighter is facing. Location is estimated using a robust localization method that employs RSSI-range models dynamically trained to cope with the peculiarities of the environment. As described in Section 4.2.1, the training and application stages of the localization method are applied simultaneously, resulting in very short delays and high adaptability. The proposed method provided localization errors around 1.4 m in the indoor experiments carried out.
- *Escape path computation*. The objective is to determine and make available to firefighters safe paths to desired locations, such as escape paths or paths to rooms with potential victims. The method adopts a guiding approach in which the path information is distributed in the WSN. Each static node keeps the information to guide the firefighters to the next adjacent room in the safe escape path. The Base Station uses optimization graph-based tools to periodically compute minimal cost paths using updated fire measurements. The new guiding information is broadcasted only if it represents a significant improvement. This functionality does not require previous building information and allows high flexibility when defining the utility to minimize. For instance, it is possible to consider accumulative effects or the influence of risks from adjacent rooms. The method is efficient and can be used simultaneously by as many firefighters as necessary.

**Figure 1.**
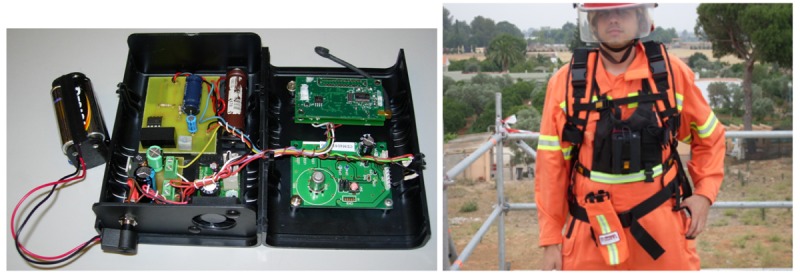
(**Left**) Prototype of the mobile nodes used in the experiments; (**Right**) Photograph taken during the validation experiments.

The proposed system and functionalities were integrated in a prototype that was validated in experiments in close-to-operational conditions. Figure 1 (left) shows a prototype of the mobile nodes used in the experiments. Each firefighter carried one mobile node located on his chest, integrated in the costumes. Figure 1 (right) shows a photograph taken during the validation experiments.

## 4. Fire and Firefighter Monitoring

### 4.1. Fire Monitoring

The objective is to keep at the Base Station updated information on the status of the fire. Measurements of the current fire and smoke status are critical to plan fire fighting. In fact, smoke inhalation is the main death cause among fire victims. Poisoning due to toxic components or small solid particles clogging the pulmonary alveoli can result in victim suffocation.

All nodes are equipped with suitable sensors to measure temperature and concentrations of *CO*_2_, *CO*, hydrogen and hydrocarbon gases. *CO*_2_ is one of the main products of combustion and it is dangerous in high concentrations. *CO* is a colorless and odorless very toxic gas produced by partial combustion when there is not enough oxygen to produce *CO*_2_. In addition, in many cases, such as industrial fires, special attention should be devoted to the monitoring of highly flammable gases such as hydrogen or hydrocarbon gases. In the system prototype implementation, we selected *Figaro TGS4161* and *TGS5042* models as *CO* and *CO*_2_ sensors, respectively. Also, we used model *Figaro FCM6812* sensor, which provides an estimation of the flammable gas concentrations adding the contribution of hydrogen, methane, butane and propane.

**Figure 2.**
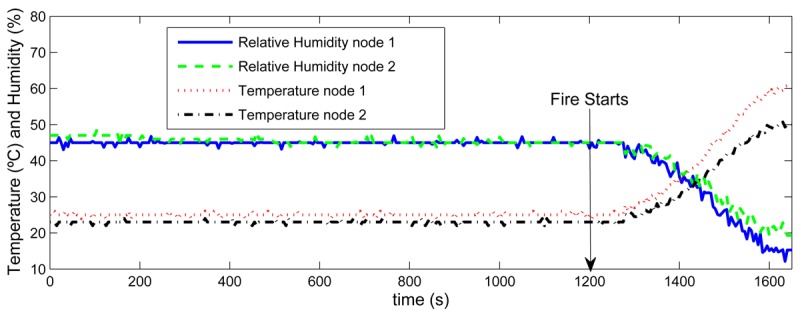
Temperature and relative humidity measurements obtained from two nodes at 3 m and 5 m from a fire machine.

Sensors selected for our system are suitable for integration due to their low size and consumption. However, they should be calibrated to improve accuracy. The gas concentration sensors were calibrated using a high performance gas measurement equipment *TESTO T350 XL*, capable of accurately measuring the concentrations of *CO*, *CO*_2_ and hydrocarbon gases, among others. The surface-mount device (SMD) temperature sensor of *TelosB* nodes was also tested. Temperature measurements of two nodes at 3 m and 5 m from a fire machine were registered, see Figure 2. Temperature raised and relative humidity dropped as soon as the fire started at time *t* = 1,280*s*.

Each node periodically gathers measurements from its sensors and forwards them in *FireSense* packets to the Base Station using dynamic multi-hop WSN routes. The information contained in these packets includes a packet identifier, an identifier of the sender node and the concentrations in parts per million of *CO*, *CO*_2_ and hydrocarbon gases. Measurement and packet transmission rates are configurable. High frequencies can improve monitoring updating and compensate packet loss, but also can increase network traffic. In the experiments carried out, packet transmission was set in the range 5–15 s, sufficient to cope with indoors fire spread rates [18]. For efficiency and scalability, *FireSense* packets use unconfirmed transmission. We prefer losing some data packets rather than adopting confirmed transmission protocols that can greatly increase network traffic with packet acknowledgments and retransmissions.

*FireSense* packets are routed to the Base Station using multi-hop channels computed using the widely proven Collection Tree Protocol (CTP) algorithm [19]. CTP is an efficient multi-hop routing protocol for transferring data from one or more sensors to one or more root nodes. CTP relies in Link Quality Estimators to dynamically select the route and can quickly adapt to the changes in the network. Adaptability is particularly interesting in our problem, where node failure is not unusual. Also, CTP is efficient in the sense that it includes mechanisms that do not require too much communication among the nodes. Notice that the CTP can start while the nodes are still being deployed, which minimizes delays.

### 4.2. Firefighter Monitoring

The objective is to keep at the Base Station the updated measurements of the firefighter locations and the conditions the firefighters are facing. Each firefighter carries a mobile node equipped with the aforementioned sensors. The Base Station registers not only the instantaneous conditions but also the accumulative gas concentrations. Thus, the participation of each firefighter in a fire episode can be managed considering the accumulative gas concentrations they have been exposed to. Also, having the updated firefighter location is critical to improve their safety. Localization in indoor environments is not a solved problem. GPS fails and methods based on Received Signal Strength Indicator (RSSI) face problems related to reflections and other interactions of radio signal with the environment. To reduce these effects, we adopt a training-based method in which each static node computes its own RSSI-range model. Then, these trained models are employed to estimate the localization of the mobile nodes.

Each mobile node periodically computes its location and gathers measurements from its sensors and inserts them in *FiremanSense* packets to be transmitted to the Base Station through the static WSN. The information contained in these packets includes a packet identifier, an identifier of the sender node, the *X* and *Y* coordinates of the mobile node and the concentrations in parts per million of *CO*, *CO*_2_ and hydrocarbon gases. Each mobile node dynamically selects the static node with which it currently has the best link quality, e.g., that with the best RSSI. The mobile node will forward data packets to that static node, which will route them to the Base Station using the multi-hop channels computed by CTP. Firefighter sensor measurement and packet transmission rates are configurable. For efficiency, the transmission of *FiremanSense* packets is also unconfirmed. Firefighter monitoring measurements are considered more critical than fire monitoring data. To reduce the impact of loss of *FiremanSense* packets in firefighter monitoring, *FiremanSense* transmission rate was set higher than *FireSense* packets.

#### 4.2.1. Training-Based RSSI Localization

A high number of RSSI-based localization methods have been developed in WSN. Range-based methods, such as multilateration [20], least squares solution [21] or maximum likelihood [22], use RSSI measurements to estimate distance to anchor nodes. Range-based methods operate well in outdoors. In indoors RSSI is highly affected by radio reflections and other interactions resulting in poor localization performance. Range-free methods, such as ROC-RSSI [23] or APIT [24], overcome these drawbacks relying on geometric considerations. Although more robust to variations in the radio channel, their localization errors are usually higher than in range-based methods. Also, some methods, such as fingerprinting [25], compare RSSI measurements with a previously computed RSSI map. These techniques often provide good results even in indoors environments but require detailed previously-computed RSSI maps of the environment and thus cannot be applied in our rapid-deployment scheme.

The proposed system adopts a training-based approach. In the training stage, each static node computes a RSSI-range model that adapts to the particularities of the environment. In the application stage, the trained models are used to locate mobile sensors using a simple but effective technique. The method combines the accuracy of range-based methods and the plasticity of training-based systems.

##### RSSI-Range Model Training

The RSSI-range model has been extensively studied in the literature [26,27]. It can be modeled as a linear relationship between the RSSI and the logarithm of the distance *D*:

(1) RSSI(D)=alogD+b

where *a* and *b* are the model parameters. Assuming a set of measurements {(*RSSI_k_*, log *D_k_*)} the model can be fitted by performing a simple linear regression:

(2) $$a=\frac{\sum_{k}\text{RSS}I_{k}\log D_{k}-\bar{\text{RSSI}}\sum_{k}\log D_{k}}{\sum_{k}{\left(\log D_{k}\right)}^{2}-\bar{\log D}\sum_{k}\log D_{k}}$$

(3) $$b=\bar{\text{RSSI}}-(a\bar{\log D})$$

where $\bar{\text{RSSI}}$ and $\bar{\log D}$ stand for the mean of *RSSI_k_* and log *D_k_*, respectively.

The training stage is as follows. Once deployed, any static node *i* starts periodically broadcasting *RegrRequest* packages. If static node *j* receives this package, in response it transmits a *RegrResp* packet containing its ID and its location *L_j_*. Node *i* receives the *RegrResp* packet and measures its RSSI, *RSSI_ij_*. It also extracts *L_j_* and computes log ‖*L_i_* − *L_j_*‖. Recall that each node knows its own location. Hence, node *i* obtains a measurement (*RSSI_ij_*, log *D_ij_*). If node *i* has sufficient measurements, it can fit its RSSI-range model *RSSI_i_*(*D*) using Equations (2) and (3). Only the *K* most recent measurements are used in order to allow model adaption to changing conditions. As node *i* gathers more measurements it periodically recalculates *RSSI_i_*(*D*).

RSSI measurements contain significant noise level that can affect the model training. Regression can cope with Gaussian noise but performs badly with non-Gaussian occasional highly noisy measurements. The proposed method adopts the RANSAC (RANdom SAmple Consensus) algorithm [28] to filter out highly noisy measurements—outliers. RANSAC is an efficient method to estimate parameters of a mathematical model from a set of measurements that can contains outliers. It requires a set of measurements {(*RSSI_k_*, log *D_k_*)} and the parameterized model in Equation (1). RANSAC iterates by selecting a random subset of the measurements as hypothetical inliers. At each iteration the model is fitted to the hypothetical inliers using Equations (2) and (3). Then, the rest of the measurements are tested against the fitted model: if an observation fits well to the estimated model, it is also considered as a hypothetical inlier. If a sufficient number of measurements have been classified as hypothetical inliers, the estimated model is considered good and the model is re-estimated using all hypothetical inliers. Finally, the model is evaluated by estimating the error of the inliers relative to the model. This procedure is repeated a number of times, each time producing either a rejected model when too few measurements are classified as inliers or a refined model together with its error measure. After the iterations, the model with lower error is selected.

**Figure 3.**
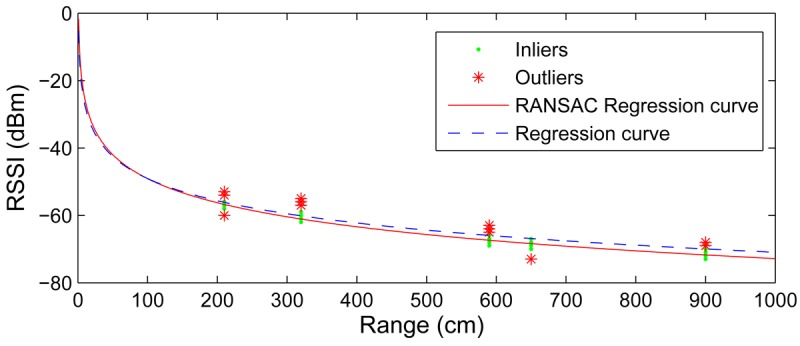
Experimental RSSI-range models obtained with and without RANSAC.

Figure 3 shows the model obtained with and without using RANSAC in an experimental test. It shows 200 RSSI measurements taken by one static node from 5 surrounding nodes. Although the RSSI measurements from each node showed low variability, the model fitting error is significant if all the measurements are considered. The RANSAC algorithm was applied. Most of the measurements fitted well to the resulting model (inliers, represented with dots), but there were some with high fitting errors (outliers, represented with asterisks). The model obtained without RANSAC, using all measurements, was *a* = −9.51 and *b* = −5.34, see Figure 3 in dashed line. The mean fitting error was 2.23 dBm. The model obtained with RANSAC, using only inliers measurements, yielded *a* = −10.302 and *b* = −1.678, with a mean fitting error of 0.49 dBm, see Figure 3 in solid line. Despite the low differences between the model parameters, the differences between the models and their impact on localization errors are significant, as will be presented in Section 6.

Notice that each static node needs measurements from only two static nodes to compute its RSSI-range model. Of course, the model trained with few measurements could have poor accuracy, but it will be improved as the number of measurements increases. Section 6 evaluates the impact of model training in localization errors as the number of measurements increases.

**Figure 4.**
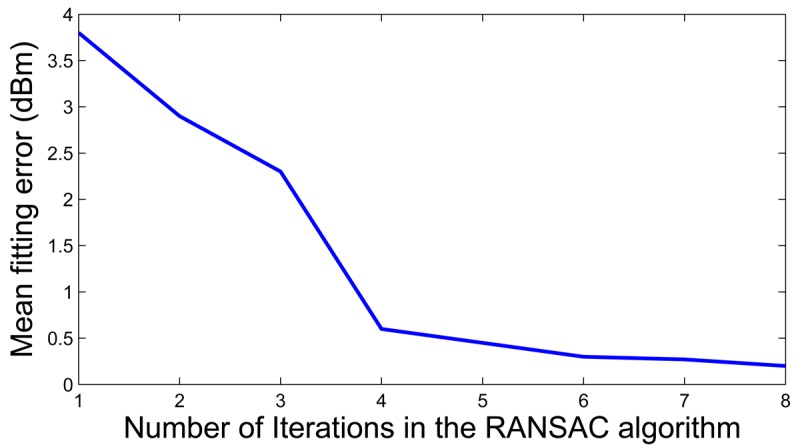
Mean model fitting error assuming different numbers of RANSAC iterations.

Model training is executed by each static node. Equations (2) and (3) can be efficiently computed in off-the-shelf WSN nodes. Keeping on with the example in Figure 3, Figure 4 shows the mean fitting error assuming different numbers of RANSAC iterations. Four iterations are enough to obtain a good fitting and no significant improvement is obtained with more. The proposed model fitting assumes 5 RANSAC iterations. It was implemented in NesC under TinyOs and its execution requires less than 170 ms in TelosB nodes.

##### Localization Algorithm

Localization uses the RSSI-range models computed in the training stage. When a mobile node *i* wants to measure its location, it broadcasts a *LocRequest* packet. Any static node *j* that receives the packet measures its RSSI and, using its RSSI-range model, estimates *D_ij_*, its distance to the mobile node *i*. Then, node *j* sends to node *i* a *LocResp* packet containing *D_ij_* and its location, *L_j_*. Static nodes responding to node *i* are considered anchor nodes for localization. Therefore, the mobile node receives a set of measurements {(*L_j_*, *D_ij_*)} from nearby anchor nodes. With these measurements it can compute its location *L_i_* applying a localization method.

Various methods such as multilateration [20] or least squares [21] can be used to provide an optimal mathematical solution to over-determined linear systems. These methods give good results when RSSI measurements have low noise levels. Low noise in RSSI measurements can cause high differences between the estimated and the actual distances. The Weighted Centroid Localization method (WCL) [29] exhibits high robustness against noise. [29] demonstrated that although Least Squares (LS) is optimal with noiseless RSSI measurements, the performance of WCL is better than LS with realistic levels of noise. We confirmed experimentally the results and adopted the WCL method. The mobile node *i* computes its location using the expression:

(4) $$L_{i}=\frac{\sum_{j=1}^{n}\left(\omega_{ij}L_{j}\right)}{\sum_{j=1}^{n}\omega_{ij}}$$

where *n* is the size of the data set and *ω_ij_* are weighting factors that depend on the distance:

(5) $$\omega_{ij}=\frac{1}{{\left(D_{ij}\right)}^{p}}$$

where *p* is an exponent to modify the influence of distance in the weights. Higher *p* gives more relevance to measurements from nearby static anchor nodes. RSSI-range models become flat—*i.e.*, insensitive to range—as range increases. The measurements from distant anchor nodes provide less useful information and are more affected by noise.

**Figure 5.**
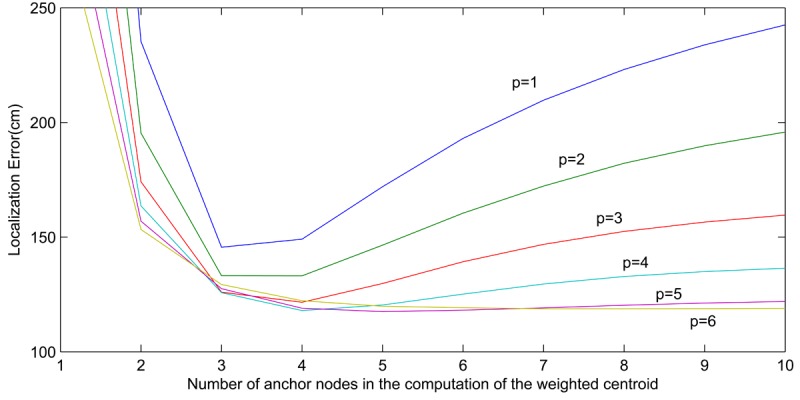
Evaluation of the effect of *p* and the number of anchor nodes in the WCL method.

We performed experimental tests to determine *p*. The setting was 21 TelosB static nodes deployed in a regular square grid. Distance between neighbor nodes was 5 m. The mobile node was put at different locations and the proposed localization method was applied with different values of *p*. The number of anchor nodes, *m*, used in the localization method was also analyzed. Figure 5 shows the mean localization errors obtained. A minimum of three anchor nodes were necessary to obtain reasonable localization errors. The results also revealed that taking into account distant anchor nodes frequently perturbs the localization performance. As above discussed, measurements from distant anchor nodes often include low information and high noise. In fact, with high values of *m*, mean localization error decreases as *p* increases since high *p* penalizes contribution of distant nodes. This experiment also analyzed the influence of *p*. The lowest localization errors were obtained with *m* = 4 and *p* = 4 and with *m* = 5 and *p* = 5. These errors were significantly lower—15%—than with *p* = 1. Although the result depends on the setting, the trends and conclusions were observed in the different settings analyzed. In the experiments shown in Section 6, where dense WSN are assumed, we selected *p* = 4.

## 5. Dynamic Firefighter Guiding

The objective is to determine the safe paths to desired locations, such as escape paths, and make them available to the firefighters. Various emergency path computation methods have been reported in the literature. In the simplest methods, when the firefighter wants to exit from a building, it broadcasts a packet requesting the escape path. The packet is sent to a decision center that computes the escape path and sends it to the mobile node. Delays and packet loss in multi-hop channels are the main disadvantages. As already discussed, the distributed guiding methods require having local information of the building and cannot be applied in our problem. The proposed guiding method adopts a hybrid approach. The computation of escape paths is carried out at the Base Station, which keeps updated information of fire/smoke status. On the other hand, the guiding information is distributed within the WSN. Each static node only keeps the information to guide the firefighter to the next adjacent room in the safest escape path.

The operation of the proposed method is as follows. When a firefighter wants to escape, it triggers the Escape Guiding Functionality in its mobile node. Then, the mobile node starts periodically broadcasting *Escape Request* packets at a high rate, 500 ms in the experiments. All static nodes that listen the packet immediately transmit a *EscapeResp* packet in response to their local guiding information. A count field is included in both *EscapeRequest* and *EscapeResp* packets so that mobile nodes can discard potential old *EscapeResp* packets. The mobile node receives the packets and extracts the local guiding information. If the mobile node receives packets with contradictory escape path information, a simple method is applied to determine which is more reliable. The mobile node keeps broadcasting until the Escape Guiding Functionality is deactivated. The guiding information reaches the mobile node in only one hop, leaving the rest of the network unperturbed: the method can be used simultaneously by many firefighters. The guiding information stored in each node requires few bytes and is independent of the size of the WSN.

### 5.1. Computation of Escape Paths

The objective is to compute escape paths that minimize risk using the measurements available at the Base Station. The proposed method adopts a graph approach: the building is modeled as an undirected graph where the vertices are rooms and the edges are connections between rooms. The outside of the building is also modeled by one vertex (ID = 0): the graph model naturally allows buildings with several exits. The cost of each edge is calculated following a cost model that uses the current fire measurements. Our objective is to compute the minimum cost path from each building room to the exit.

Graph representations are suitable in our problem since many graph-based optimization algorithms have been developed. Two main variations of the shortest path problem can be found: the single-source shortest path problem, which finds shortest paths from a source vertex to all other vertices in the graph; and the all-pairs shortest path problem, which finds the shortest paths between every pair of vertices in the graph. In our problem, the objective is to compute the shortest path to the exit: it is a single-source problem. There are two main algorithms to solve this problem, the Bellman-Ford algorithm and the Dijkstra algorithm. We selected Dijkstra [30] since it is faster, intuitive and easy to implement.

Dijkstra solves the single-source minimum cost path problem for a graph with nonnegative edge costs. It obtains the minimum cost path from an initial vertex to the rest of the graph vertex, producing a minimum cost tree. The outside of the building (vertex 0) is taken as the initial vertex. Dijkstra computes the minimum cost paths from the vertex 0 to each of the rooms. Since the building graph is undirected, these will be as well the minimum cost paths from each of the rooms to the vertex 0. Each resulting path is an ordered list of contiguous vertices—room identifiers—that ends in vertex 0. Paths can be seen as a concatenation of local guiding commands. The local guiding information of node *i*, {*LGI_i_*}, is represented by a pair with the current room identifier and the next room in the path.

The proposed method requires an edge cost model. The cost of the edge between vertices *i* and *j*, *C_ij_*, is related to the conditions a firefighter are exposed to in its way from room *i* to *j*. It is not the objective of this paper to define an accurate cost model. Expertise from firefighter is critical here. In this paper, we used a simple but efficient model that illustrates the capabilities of the approach. In the cost model used *C_ij_* is considered as the sum of three terms: *C_ij_* = *CA_ij_* + *CB_ij_* + *CC_ij_*. The first one, *CA_ij_*, refers to cases in which a strong risk is identified, such as high temperatures or flammable gases in a room. Presence of a strong risk in rooms *i* and/or *j* is modeled setting *CA_ij_* to very high values. Otherwise, it is set to zero. The objective is to prevent these rooms from being included in escape paths.

The second one, *CB_ij_*, refers to the presence of accumulative risks, such as toxic gases. Humans can tolerate low concentrations or short expositions until a certain threshold is reached. Its objective is to minimize the exposition to toxic gases. *CB_ij_* is computed summing the contributions of both rooms:

(6) $$CB_{ij}=CB_{i}d_{i}+CB_{j}d_{j}$$

where *d_i_* is the distance between the center of room *i* and the door between both rooms and *d_j_* is the distance between the door and the center of room *j*. *CB_i_*, the accumulative risk in room *i*, can be computed summing the contributions of all toxic gases considered:

(7) $$CB_{i}=\sum_{k}\left(\alpha_{k}C_{k,i}\right)$$

where *C_k,i_* is the concentration of toxic gas *k* in room *i* and *α_k_* is the weighting factor to consider the gas toxicity. In the experiments shown in Section 6, *CO* and *CO*_2_ were considered with weights *α*_1_ = 0.7 and *α*_2_ = 0.3, respectively, for the highest toxicity of *CO*. The third term, *CC_ij_*, is originated by the risk of being inside a building in flames. Its objective is to minimize the length of the escape path. *CC_ij_* is proportional to *d_i_* + *d_j_*.

This cost model can be modified to include more elaborate cases. For instance, in case of detecting strong risks such as high concentrations of flammable gas in one room, high cost can be assigned not only that room but also the adjacent rooms. Also, *C_ij_* could include a term to consider the cost of contiguous rooms. The model adopted is simple and efficient and showed good performance in the experiments carried out. However, to derive a model to be used in the real application, firefighter advices and more experiments are necessary.

Next section describes the routing of the guiding information from the Base Station to the static nodes.

### 5.2. Routing of Local Guiding Information

The objective is to deliver local guiding information computed at the Base Station to each static node. The solution adopted follows a flooding approach modified to reduce the number of retransmissions. Nodes with no *children nodes* in the CTP tree do not retransmit the flooding packets. Each node *i* keeps a list of its children. When it receives a data packet, it registers the sender ID in its children list. To cope with changes in the tree, there is a timeout for each node in the children list. If node *i* receives a data packet from node *j* it resets its timeout. If the timeout expires node *j* is deleted from its children list.

The Base Station periodically computes updated local guiding information for the WSN nodes ({*LGI*}). Each version of updated {*LGI*} is tagged with a sequence number *SeqF*. When the Base Station has new local guiding information, it inserts it in *m GuidingInfo* packets containing {*LGI_i_*}, *SeqF* and the number of the packet in this flooding, see Table 1. Then, the packets are broadcasted. Each static node in the tree keeps a list of the *GuidingInfo* packets if has received and their *SeqF*. When a node *k* receives the *GuidingInfo* packet it checks if he has received that packet previously. In that case, the packet is ignored, otherwise it compares *SeqF* of the packet with the sequence number of the last *GuidingInfo* packet it received. If *SeqF* is higher and the packet contains information for him (*LGI_k_*), it updates its local guiding information and updates the version *SeqF*. Then, node *k* checks if its children list is not empty. In that case it broadcasts the *GuidingInfo* packet, or ignores it otherwise.

The Base Station triggers a flooding only if the new guiding information is significantly better than the information that is actually in the static nodes. The Base Station keeps track of the version *SeqF* of the local guiding information used by each node. The *FireSense* packets used in Section 4 include a field in which static nodes report the version of the local guiding information *SeqF* each node is using. With this information, the Base Station can simulate the behavior of the actual escape paths. If the performance difference-cost-between the escape paths computed with the new guiding information and those computed using the actual information is above a threshold, the Base Station triggers a new flooding. This mechanism allows monitoring the guiding information that is actually in the WSN, minimizing the number of flooding.

## 6. Experiments

### 6.1. Partial Experiments

#### 6.1.1. Training-Based RSSI Localization

This section evaluates the accuracy of the proposed localization method, its robustness against misplacement of static nodes and its performance during the training stage.

##### Accuracy

**Figure 6.**
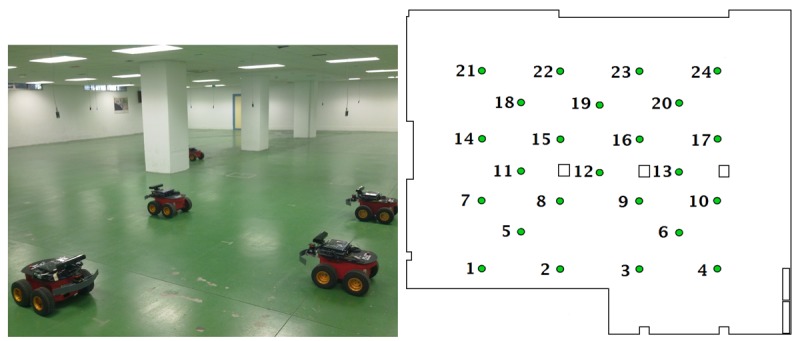
(**Left**) Picture of the *CONET* testbed; (**Right**) Scheme of a typical WSN deployment in the testbed room.

The experiments were carried out in the *CONET Integrated Testbed* [31], see Figure 6 (left). The testbed (*http://conet.us.es*) is a remote open tool to assess and compare multi-robot and WSN methods. The testbed deployment includes a configurable static WSN with nodes hanging from the ceiling. Figure 6 (right) shows a typical setting. The testbed also includes five *Pioneer AT* robots. Robots carrying mobile nodes are convenient tools in localization experiments. They have greater motion accuracy than humans and the same experiment can be performed hundreds of times with high repeatability. The testbed is equipped with components that provide the ground truth location of the robots. Each robot is physically connected to one WSN node—mobile node—through a serial line. A simple protocol allows robot-WSN bidirectional interchange of data, requests and commands. The testbed is installed in a 500 m^2^ room in the School of Engineering of Seville.

A set of 25 localization experiments was performed. In the training stage, each static node obtained its RSSI-range model. In the application stage the robots followed a pre-assigned trajectory while their onboard mobile node broadcasted *LocRequest* packets. In response each mobile node received *LocResp* packets from surrounding static nodes and computed its current location. Each mobile node transmitted its computed location to its robot. The robot logged in a synchronized way the estimated location together with the ground truth location of the robot.

**Figure 7.**
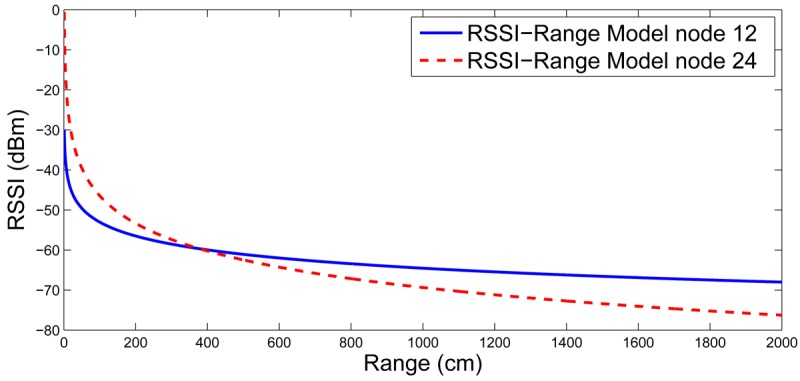
RSSI-range model for two nodes of the *CONET* testbed.

**Figure 8.**
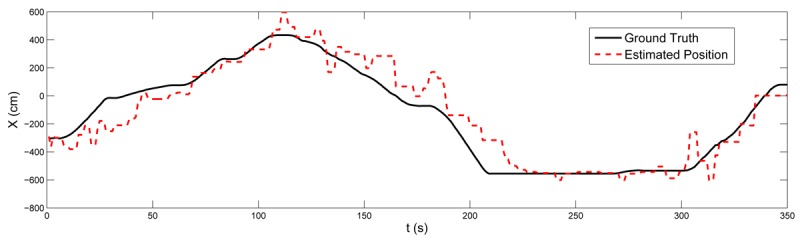

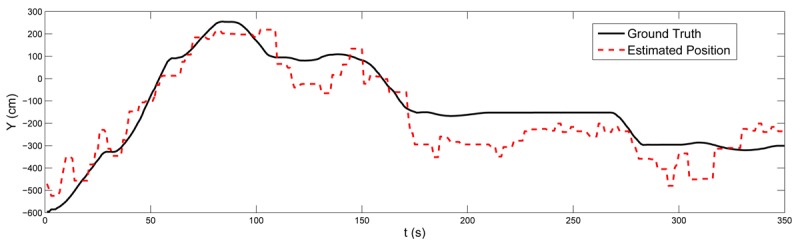
Results of the proposed localization method in axes *X* (up) and *Y* (bottom).

Figure 7 shows the RSSI-range models obtained in the training stage by nodes 12 (solid line) and 24 (dashed). Differences between their radio-chip units, antenna orientations and their surroundings, among others, resulted in different RSSI-range models. Figure 8 shows the location in axes *X* (top) and *Y* (bottom) obtained in one experiment. The estimated location is represented in solid line and the ground truth is in dashed line. The mean localization error was 124.43 cm and its standard deviation was 68.09 cm. The mean errors in axes *X* and *Y* were 75.55 cm and 49.56 cm, respectively. These results depend on the setting. We repeated the experiments with settings (rhombus grids, random deployment) and the results were satisfactory as well. This experiment was also used to evaluate the impact of *RANSAC* outlier rejection. In the training stage *RANSAC* was substituted by the simple regression in Equations (2) and (3). In this case, the mean localization error was to 141.56 cm, 13% higher. As expected, the rejection of outliers improves the localization accuracy. The same experiment was repeated using LS instead of WCL. In this case, the mean localization error was 167.44 cm—34% higher than WCL—and the standard deviation was 103.84 cm. Although LS is mathematically optimal, its high sensitivity to noise in RSSI measurements degrades its performance in real situations.

##### Robustness to Static Nodes Misplacement

**Figure 9.**
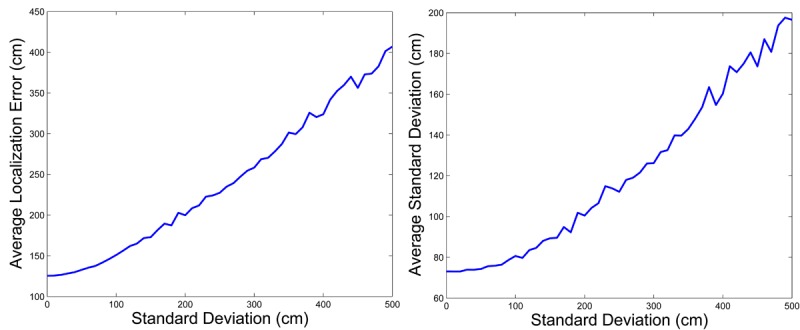
Impact of anchor nodes misplacement in mean localization error (**left**) and standard deviation (**right**).

In our approach, static nodes are deployed by firefighters. There can be errors between the planned locations and where the firefighter actually put the nodes. Misplacement errors affect the training and the application stages. This test analyzes the impact of misplacement of static nodes. We simulated the localization method using four anchor nodes. The *X* and *Y* coordinates of each anchor node were distorted with zero mean Gaussian noise with standard deviations in the range from 0 to 500 cm. In total 500 samples for each standard deviation were taken. Notice that with *σ* = 500 cm, the 99% of the noise samples are in the range [−14.5, 14.5] m. We believe that these misplacement errors are lower than those usually found in the real problem. The RSSI measurements were assumed with a realist noise level. Then, we executed the training and application stages of the method and registered the localization errors. The mean localization error assuming no misplacement was 128 cm and the standard deviation was 73 cm, due to the noise assumed in the RSSI measurements. The results in Figure 9 show that the localization mean error and its standard deviation behave fairly well with misplacement errors. When the standard deviation of the misplacement errors increases from 0 to 500 cm, the mean error only increases from 128 to 407 cm and its standard deviation increases from 73 to 196 cm, which illustrates the good behaviour of the proposed method with misplacement of anchor nodes.

##### Performance During the Training Stage

As pointed out in Section 4.2.1, the RSSI-range model can be estimated using measurements from only two static nodes. Once an initial model has been computed, it can be applied to localize mobile nodes. Thus, the training stage can be simultaneous with the application stage. Each static node keeps receiving new training measurements and as soon as it re-calculates a new model, it can be used in the application stage. At the beginning, RSSI-range models computed with few measurements will not achieve high localization accuracy. With more measurements, the trained models will be more and more precise, resulting in higher accuracy. The objective of these experiments is to evaluate the evolution of the localization accuracy during this process.

The experiments were carried out as follows. Robots carrying a node followed their trajectory in the environment. At a certain time, both the training stage and the operation stage started at the same time. As soon as a static node had gathered enough RSSI measurements, it built its own RSSI-range model. As the number of RSSI measurements gathered increased, the node recalculated its model. Meanwhile, mobile nodes broadcasted *LocRequest* packets. If a static node received a *LocRequest* packet but still had not built its RSSI-range model, it ignored the packet. Only static nodes that had their own RSSI-range model responded. With the responses, mobile nodes estimated their location. In this experiment, the localization method was applied using only the measurements—and the RSSI-range models—from four static nodes. The objective is to evaluate the impact of the accuracy of these four RSSI-range models on the localization errors.

**Figure 10.**
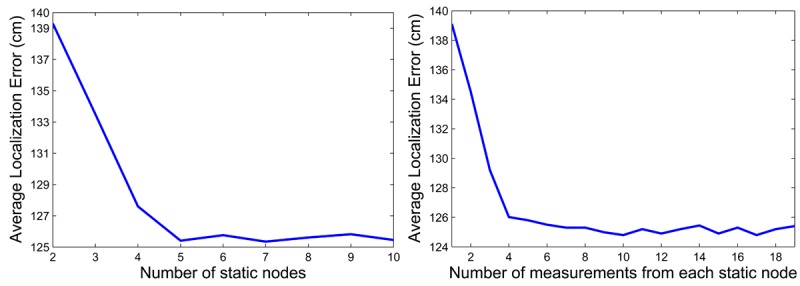
(**Left**) Average localization error *VERSUS* number of static nodes taken into account in the training of the RSSI-range models. Each static node contributed with 200 measurements to the training set; (**Right**) Average localization error *VERSUS* number of measurements from each static node taken into account in the training of the RSSI-range model. The training set contains measurements from five different static nodes.

First, the impact of the number of static nodes considered in the training was evaluated. Each static node contributed with 200 measurements to the training set. Figure 10 (left) shows the localization errors obtained. If the models were trained considering two static nodes, the average localization error was 139.4 cm. As expected, when the number of static nodes increased—better trained models—the localization error improved: 125.2 cm using models that were trained with five static nodes. In addition, there was not a significant improvement if more than five static nodes were considered in the training.

Then, we evaluated the impact of the number of measurements each static node contributes to the training set. The training set contained measurements from five different static nodes. Figure 10 (right) shows the localization errors obtained. If the RSSI-range models were computed using only one measurement from each static node—a total of five measurements in the training set, the average localization error was 138.4 cm. The localization error was 125 cm if models are trained with five measurements from each static node, *i.e.*, training set with 125 measurements. Again, there was not a significant improvement when the number of measurements of each static node was higher than four: it is not necessary to take many measurements from each static node to train RSSI-range models.

The above analyzes conclude that initial RSSI-range models computed using few measurements from few static nodes can provide sufficient accuracy. Accuracy improves as the models are refined with more measurements but there is not a significant improvement when the number of measurements is above a threshold. This validates the proposed localization approach in which the training stage and the application stage are simultaneous.

#### 6.1.2. Dynamic Firefighter Guiding

**Figure 11.**
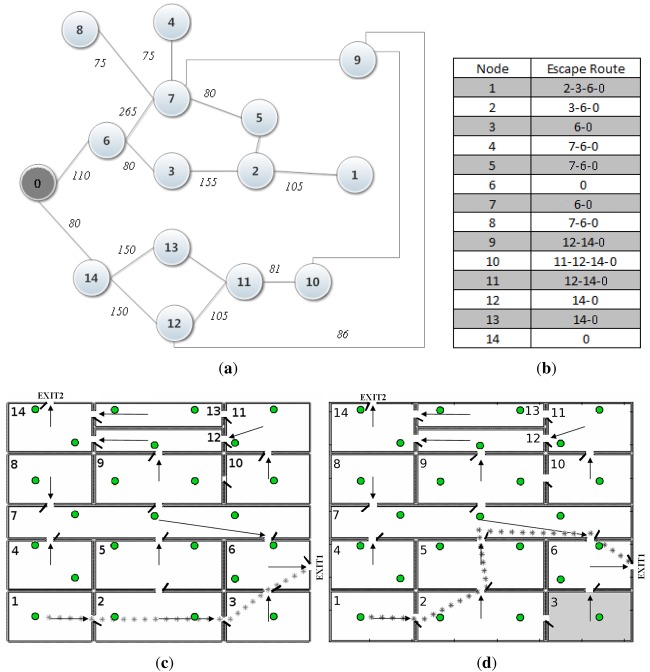

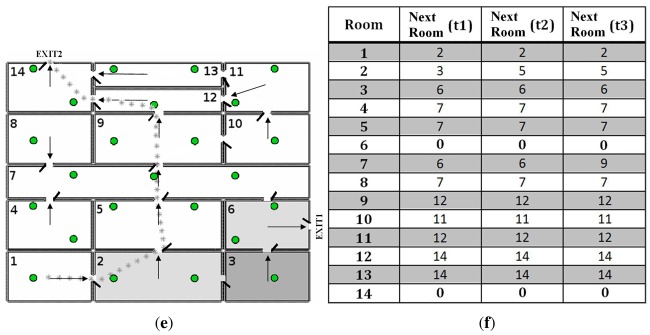
Dynamic firefighter guiding experiments carried out in the *CONET* testbed: (**a**) building graph; (**b**) optimal paths at *t*_1_; (**c**–**e**) maps with resulting escape paths at times *t*_1_, *t*_2_ and *t*_3_; (**f**) local guiding information at times *t*_1_, *t*_2_ and *t*_3_.

This section experiments the proposed firefighter guiding method in a real setting. The experiments were carried out in the *CONET* testbed. The building map used is shown in Figure 11(c). The WSN nodes in the *CONET* testbed are represented as circles. With this map, all rooms include at least one node and some rooms have up to three nodes. The severity of fire conditions is represented by the color of each room: darker colors mean worse conditions. The fire measurements used in the experiment were not real: they were values previously loaded in the nodes' memory. The radio transmission power of WSN nodes was reduced to allow multi-hop channels. The building can be modeled by the graph in Figure 11(a). Each vertex represents a room. Vertex 0 corresponds to the outside of the building. Each edge is associated to a cost computed with the model used in Section 5.

The performance of the method with three different fire conditions was tested. At time *t*_1_ the sensors detected no fire alarms. In this case, the shortest paths are considered optimal. The optimal escape paths are in Figure 11(b). Each path is an ordered list of room identifiers. The last identifier is 0, the building outside. The optimal path for room 1 is {2, 3, 6, 0}. Each path can be seen as a concatenation of local guiding information containing the current room and the next room in the path. For instance, the local guiding information for the nodes in room 1 is *LGI*_1_ = (1, 2).

Then, the Base Station floods the local guiding information in the WSN in one *GuidingInfo* packet. *SeqF* was set to 1. Each static node received the *GuidingInfo* packet and uploaded the guiding information. Figure 11(c) shows with arrows the guiding information at the nodes on each room of the building.

Then, a robot equipped with a mobile node-firefighter-activated the Escape Guiding Functionality. Its mobile node started broadcasting *EscapeRequest* packets. In response, static nodes transmitted *EscapeResp* packets with guiding information. The mobile node received them and discarded packets with old count: only *EscapeResp* packets corresponding to the last *EscapeRequest* packet were considered. After this filtering, the mobile node selected one *EscapeResp* packet using simple rules that consider their RSSI and *SeqF*, the version of the local guiding information. The mobile node sent the guiding information to the robot through the serial interface. The robot moved accordingly following the escape paths.

**Figure 12.**
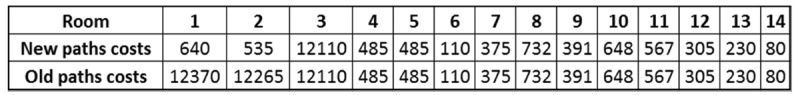
Costs of the new and old escape paths computed with the measurements at *t*_2_.

At time *t*_2_ the node deployed in room 3 sensed high *CO*_2_ concentrations. The measurements reached the Base Station, which recomputed the escape paths. Figure 12 shows the cost of the new paths and the cost of the old paths computed using the current fire conditions. The new paths achieved a significant improvement for rooms 1 and 2. Thus, new fire status recommended updating the distributing the new guiding information distributed in the WSN. Figure 11(d) shows with arrows the guiding information stored in the nodes after the new flooding. The new escape paths avoided room 3. While in *t*_1_ the optimal path for room 2 was {3, 6, 0}, in *t*_2_ it was {5, 7, 6, 0}.

After a while, *CO*_2_ spread to rooms 2 and 6. When the measurements reached the Base Station, it recomputed the escape paths. The new local guiding information was flooded. The resulting guiding information stored in the nodes after the flood at *t*_3_ is in Figure 11(e). Figure 11(a–c) shows with asterisks the resulting optimal path for room 1 in the three conditions. In *t*_1_ the optimal path was {2, 3, 6, 0}. In *t*_2_ it was {2, 5, 7, 6, 0} and in *t*_3_ it was {2, 5, 7, 9, 12, 14, 0}. The experiment was repeated 10 times with random fire conditions, and at all times the method performed as expected.

The method is rather robust to losses of *EscapeRequest* and *EscapeResp* packets. Temporal WSN disconnections can involve loss of updated fire measurements at the Base Station, which can result in unsuitable escape paths. Frequent transmission of *FireSense* packets help preventing these cases. Also, CTP re-computes multi-path routes dynamically to reduce the impact of node failures and unstable radio links. WSN disconnections can involve loss of local guiding information by static nodes. For instance, the interaction between updated and old guiding information in different nodes can originate firefighter guiding loops. Loss of local guiding information is solved by re-flooding updated packets. As said before, the Base Station keeps track of *SeqF* the version of the local information stored in each node and triggers new flooding if the actual escape paths are unsuitable. After 10,000 simulations, not a cycle was created by the proposed system. For safety, a simple guiding loop detection method for mobile nodes was devised: if a cycle is detected the mobile node warns the firefighter.

### 6.2. Validation Experiments

The proposed system has been validated in experiments in close-to-operational conditions carried out at two floors of the L1 Laboratories building of the School of Engineering of Seville. There are two stairs between both floors, each of them is modeled with a vertex in the building graph. The building has two exits at the first floor. The building has no previous WSN infrastructure. The proposed system was set with the values used in Section 6.1.1. For safety reasons, the experiments were carried out with no real fire. Below the story of the experiment and some results are summarized.

**Figure 13.**
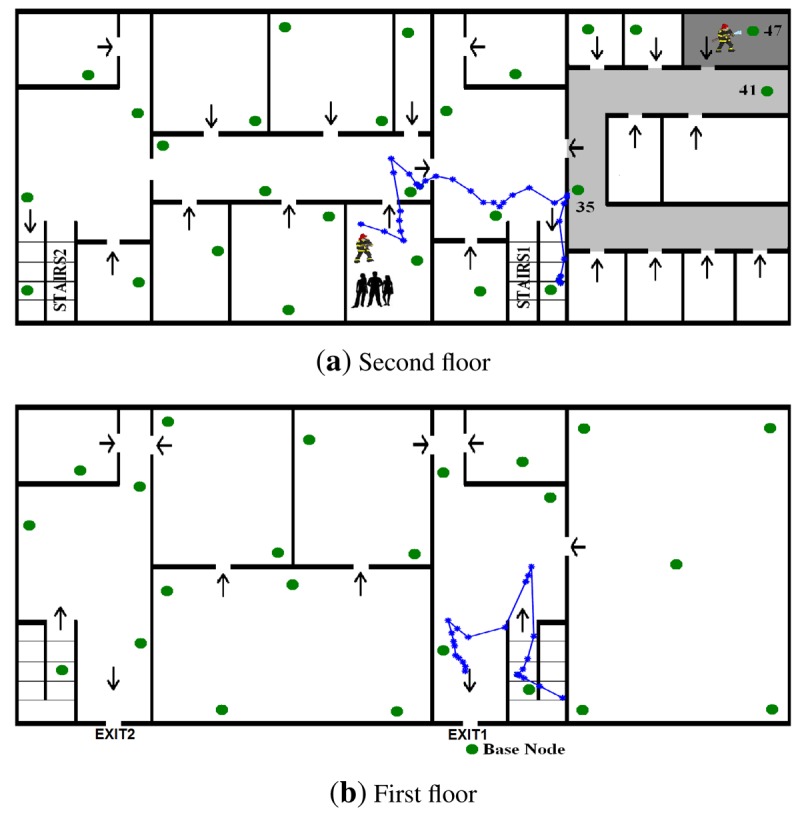
Maps of the second (**a**) and first (**b**) floors of the building with fire conditions and guiding information at time *t*_1_. The trajectory followed by FIREMAN1 while escaping from the building is also shown.

A fire was declared in the building. Soon, the fire brigade arrived. After a quick analysis, they decided the WSN deployment. Two firefighters equipped with mobile nodes entered the building and started deploying the nodes—beginning with the first floor—at assigned locations. Once deployed, each static node started dynamically creating the CTP routing channels. They also interchanged *RegrRequest* and *RegrResp* packets and obtain their RSSI-range models. After a short delay, fire/smoke measurements were received at the Base Station. FIREMAN2 detected a fire at the second floor. He stopped deployments. His finding was confirmed by nodes ID = 47, ID = 41 and ID = 35, which sensed abnormal *CO*_2_ measurements. The rest of the nodes measured normal readings. Meanwhile the Base Station computed escape paths using fire measurements and flooded the local guiding information for each WSN node. Figure 13 shows with arrows the local guiding information stored in the nodes in each room. When the firefighter activated their mobile nodes, they started broadcasting *LocRequest* packets and receiving *LocResp* packets from nearby static nodes. Each mobile node computed its own location and created *FiremanSense* packets that were sent to the Base Station. The firefighter location and measurements were visualized and registered by the Base Station.

At time *t*_1_ FIREMAN1 found a group of students in a room on the second floor. He stopped deploying nodes and activated the Escape Guiding Functionality in his mobile node. The mobile node started broadcasting *EscapeRequest* packets and receiving *EscapeResp* packets from nearby nodes. The escape path passed from the second to the first floor using STAIRS1. FIREMAN1 followed the escape path and guided the students out of the building at EXIT1. Figure 13 also shows the trajectory of FIREMAN1 estimated by the proposed localization method while the Escape Guiding Functionality is active.

**Figure 14.**
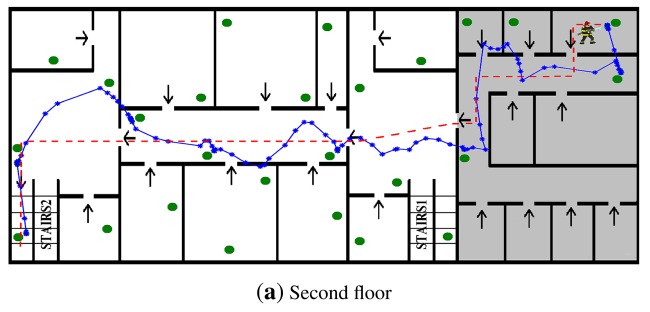

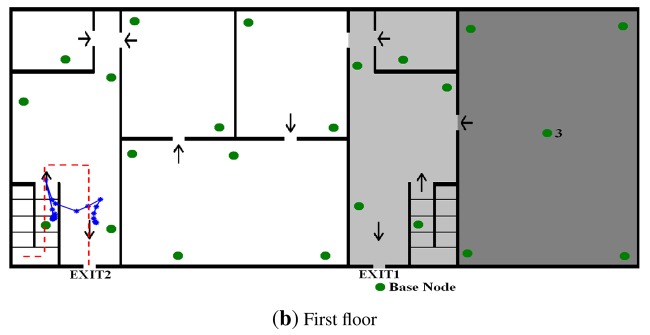
Maps of the second (**a)** and first (**b**) floors of the building with fire conditions and guiding information at time *t*_2_. The straight-line representation of the escape path (dashed line) and the actual trajectory followed by FIREMAN2 (solid line) are also shown.

**Figure 15.**
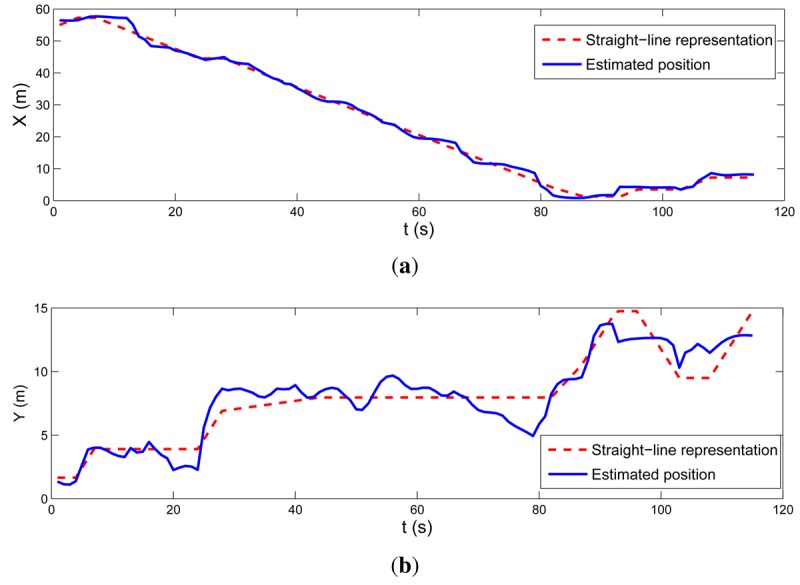
Locations in *X* (up) and *Y* (bottom) axes of FIREMAN2 location estimated by the proposed method while its Escape Guiding Functionality was active. The straight-line representation (dashed line) and the actual trajectory followed by FIREMAN2 (solid line) are shown.

After a while, node ID = 3 detected a low concentration of hydrocarbon gases, see Figure 14. The head of the fire brigade was informed that there was a Fuel Cell Unit in a room on the first floor. Then, the Base Station operator manually assigned that and the surrounding rooms with high costs in the escape path computation module. As a result, the optimal escape paths were updated and *GuidingInfo* packets with the new guiding information were flooded in the WSN. Static nodes updated their local guiding information, represented with arrows in Figure 14. At time *t2* FIREMAN2 got to extinguish the fire. To get out of the building he activated the Escape Guiding Functionality in his mobile node. In this case, the escape path used STAIRS2 and EXIT2. Figure 14 shows in dashed line the actual trajectory estimated by the proposed system while the Escape Guiding Functionality of FIREMAN2 was active. A straight-line representation of the escape path is also depicted in solid line. Figure 15 compares both (straight-line representation and actual locations) in *X* and *Y* axes.

**Figure 16.**
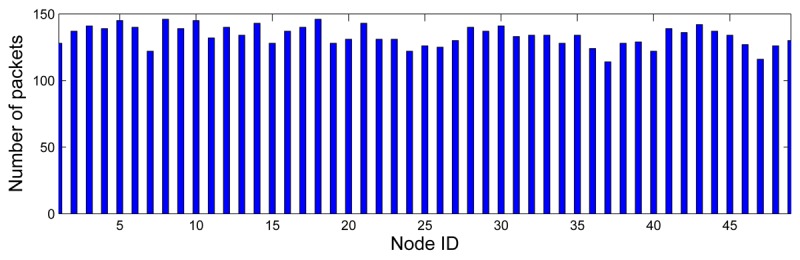
Number of FireSense packets received per node ID.

The experiment took 25 minutes. Nodes were configured to transmit *FireSense* packets every 10 seconds: each node transmitted around 150 packets. Figure 16 shows the number of *FireSense* packets from each static node that were received at the Base Station. An average of 88.7% of the *FireSense* packets arrived errorless at the Base Station. Nodes with higher ID—deeper in CTP trees—had high loss rate (up to 23.3% for node ID = 37), which was attributed to the higher numbers of retransmissions.

## 7. Conclusions and Future Work

This paper describes a WSN tool to increase safety in urban and industrial fire fighting. It uses a WSN with static and mobile nodes carried by firefighter. However, it assumes that there is no preexisting WSN in the building, which improves applicability but imposes constraints such as lack of previous information and need for high adaptability and low delays. Once deployed by firefighter, static nodes provide a communication backbone with the Base Station through dynamic multi-hop routes. All nodes are equipped with fire/smoke sensors. Measurements from static and mobile nodes are transmitted to the Base Station for monitoring and registering. This paper focuses on the following functionalities: fire monitoring, firefighter monitoring and dynamic escape path computation.

The proposed system includes a robust localization method that employs RSSI-range models dynamically trained to cope with the peculiarities of the environment. The training and application stages of the method are applied simultaneously, resulting in high adaptability. The proposed localization method exhibited significant accuracy and robustness in the indoors experiments carried out. The system also includes an escape guiding method. The Base Station uses optimization graph-based tools to periodically compute minimal cost paths using updated fire measurements. The new guiding information is broadcasted only if it represents a significant improvement. The method is efficient and can be used simultaneously by as many firefighters as necessary. All these functionalities were integrated in a prototype that was satisfactorily tested in experiments in close to operational conditions.

The proposed system showed promising results in the tests carried out. Of course, further testing and evaluation are necessary before its use in the real application. The codification of local guiding information, which can simplify interpretation by firefighter and minimize potential errors, and the development of security mechanisms are also necessary before its use in the real application. In the proposed method, each static node knows its own location. The iterative cascade-like localization of the whole WSN using few initial anchor nodes with known location is also under research. In addition, the real application should require enclosures to protect nodes from the environment. The prototype we develop used a standard plastic box but the design of the enclosure considering different cost-performance solutions is currently under work. Expensive solutions are more effective from a protection point of view. We are currently working on the evaluation of the most suitable enclosure from a cost-robustness tradeoff perspective.

## Figures and Tables

**Table 1.** *GuidingInfo* packet. Each column includes the field name and its size.

**Pkt ID**	**SeqF**	**Pkt N°**	**Node ID**	**Next Room**	…	**Node ID**	**Next Room**
1	1	1	2	2	…	2	2
